# Bypassing pleural aspiration for early tissue diagnosis in suspected pleural malignancy: Development and external validation of a predictive model using supervised machine learning

**DOI:** 10.5339/qmj.2026.14

**Published:** 2026-03-19

**Authors:** Adhnan Omar, Artemio Gonzales, Alina Ionescu

**Affiliations:** 1 Department of Respiratory Medicine, Aneurin Bevan University Health Board, Newport, United Kingdom *Email: Adhnan.Omar2@wales.nhs.uk

**Keywords:** Pleural neoplasm, machine learning, validation studies, predictive value of tests, tissue biopsy

## Abstract

**Background and objective:**

The conventional diagnostic pathway for pleural malignancy involves pleural aspiration, often followed by tissue biopsy. However, pleural aspiration has limited sensitivity, which can potentially delay a definitive diagnosis. The objective of this study was to develop and externally validate a predictive model using clinical data to identify patients who could safely bypass pleural aspiration and proceed directly to tissue biopsy.

**Methods:**

This was a retrospective cohort study of patients presenting to an acute hospital in the UK with suspected pleural malignancy between 2016 and 2025 (n = 646). A Random Forest classifier was trained on this dataset, and its performance was evaluated using an independent external validation cohort from another acute hospital in the UK (*n* = 32). Model performance was assessed using standard metrics, including accuracy, precision, recall, F1-score, and the area under the receiver operating characteristic curve (ROC-AUC).

**Results:**

The model demonstrated robust performance in the internal dataset and maintained its predictive strength during external validation (AUC: 0.86 vs. 0.83; precision 87% vs. 85%; recall 79% vs. 75%). Among the variables, computed tomography findings were the most influential predictor, as quantified by Gini importance. A simplified scoring system was subsequently derived for potential bedside clinical application.

**Conclusion:**

This externally validated model provides a valuable decision-support tool for clinicians, facilitating earlier tissue diagnosis in patients with suspected pleural malignancy. By potentially avoiding non-diagnostic aspiration, the model can streamline the diagnostic pathway and expedite patient care. Successful external validation enhances confidence in the model’s generalizability and supports its potential for implementation into routine clinical practice.

## 1. INTRODUCTION

Pleural malignancies represent a significant diagnostic challenge, typically requiring a multi-modal approach combining imaging, cytology, and histology to achieve a definitive diagnosis. While tissue acquisition remains the gold standard for confirming pleural malignancy—commonly achieved through thoracoscopy, video-assisted thoracoscopic surgery (VATS), or computed tomography (CT)-guided biopsy—the initial step in current guidelines often involves pleural aspiration.^1^ However, this procedure frequently yields non-diagnostic or inconclusive results, leading to procedural delays, repeat interventions, and increased patient morbidity.^2^ The limited diagnostic accuracy of pleural fluid cytology—particularly in the context of advanced imaging findings—has been highlighted in recent studies.^3,4^

In clinical practice, a substantial proportion of patients present with prior imaging and a clinical history that is highly suggestive of malignancy. In such scenarios, the necessity of an initial pleural aspiration becomes questionable. Recent international studies have highlighted the limited diagnostic yield of aspiration in suspected malignant pleural effusions, advocating for a more individualized diagnostic approach.^3^ There are now national guidelines for pleural disease recommending a more nuanced approach, suggesting that image-guided biopsy may be a more appropriate first-line investigation in certain circumstances.^2^ This study addresses a critical unmet need: the development of a decision-support tool that uses readily available clinical variables and CT imaging to identify patients who can safely bypass aspiration and proceed directly to definitive tissue diagnosis.

Recent advancements in artificial intelligence (AI) and supervised machine learning (ML) offer promising tools for enhancing diagnostic decision-making.^5,6^ The application of AI in medicine leverages computational power to identify patterns in vast datasets that are often imperceptible to humans. Machine learning, a subset of AI, involves training algorithms on historical data to make predictions or classifications without being explicitly programmed for every scenario. This work focuses on a specific type of machine learning, supervised learning, in which the model learns from a labelled dataset (i.e., known cases of confirmed malignancy). This approach, using algorithms such as Random Forest, enables the creation of predictive models that integrate multiple clinical factors to generate a single, risk-based output. While many clinical prediction models have traditionally relied on logistic regression, machine learning offers the advantage of capturing nonlinear relationships and complex interactions between variables, which may more accurately reflect biological and clinical reality.^7,8^

A key limitation of many AI-driven studies, however, is the lack of rigorous external validation beyond their development cohort, which compromises their generalizability to real-world clinical settings. In the present study, the authors aimed to develop and externally validate a supervised machine learning model using a Random Forest algorithm to predict the need for tissue biopsy in suspected pleural malignancy, leveraging only clinical history and CT imaging findings.

## 2. METHODOLOGY

### 2.1. Study design and population

A retrospective cohort study was conducted involving a purposive sample of 678 patients across two acute centres in the United Kingdom between 2016 and 2025. Clinical data were collected from a single centre—Grange University Hospital (GUH), Cwmbran, UK (*n* = 646)—for model development and subsequently from a separate centre—Royal Glamorgan Hospital (RGH), Ynysmaerdy, UK (*n* = 32)—for external validation. The required approvals were obtained from the GUH (SE/1688/24 on 21 October 2024) and RGH (CTM [Cwm Taf Bro Morgannwg Health Board]/2259/25 on 2 June 2025) institutional review boards in accordance with their respective guidelines.

### 2.2 Inclusion and exclusion

Included patients were those who underwent investigation and diagnosis by a multidisciplinary team comprising respiratory, oncology, radiology, and thoracic surgical specialists, and were confirmed to have pleural malignancy between 2016 and 2025. The cohort comprised both confirmed malignancy cases (positive class) and confirmed benign cases (negative class). Patients with inconclusive final diagnoses or loss to follow-up were excluded.

### 2.3 Data collection and variables

Predictor variables were extracted from the electronic health records and included the following:

- CT findings (categorized as malignant, benign, or uncertain)
- History of asbestos exposure (yes/no)
- Age at the time of presentation (continuous variable)
- Gender (coded as 1 = male, 0 = female)

Pleural aspiration results were intentionally excluded from the dataset to develop a model that could guide the need for biopsy based solely on non-invasive data. The outcome variable was the presence of malignancy confirmed by tissue biopsy. Patients with missing predictor variables were excluded from the analysis using a complete-case approach.

### 2.4. Model development: Supervised machine learning

A Random Forest classifier was used, which is a robust supervised ML technique that constructs an ensemble of decision trees and aggregates their predictions.^9^ Each tree is trained on a random subset of the data and a random subset of features. The final prediction is determined by a majority vote among the trees. This method is particularly well-suited to clinical data due to its ability to capture complex nonlinear interactions and its inherent resistance to overfitting.

The model was developed using scikit-learn in Python.^7^ The dataset was randomly partitioned into training (80%) and testing (20%) subsets. The target variable was tissue-confirmed malignancy. Model performance was assessed using standard classification metrics, including accuracy, precision, recall, F1-score, and ROC-AUC. Precision and Recall were calculated using the following equations:

   Precision = TP/(TP+FP)

   Recall = TP/(TP+FN)

where TP represents the number of true positives, FP the number of false positives, and FN the number of false negatives.

Default hyperparameters were used, with 100 estimators. The performance of the final model was evaluated on the independent testing set.

### 2.5. Model calibration

To assess calibration, a calibration curve was generated by plotting predicted probabilities against observed outcomes. Binning was used to visualize the agreement between predicted and actual cancer probabilities, and the Brier score was computed to quantify calibration performance.^8^

### 2.6. External validation

The final trained model was applied to an independently collected external dataset. No retraining or parameter tuning was performed on the new data, ensuring true external validation. The same performance metrics were evaluated in the external cohort to assess the model’s generalizability.

### 2.7. Clinical score derivation

A simplified scoring system was derived from the most predictive features based on their Gini importance in the Random Forest model.^10^ Gini importance measures the extent to which a feature contributes to reducing impurity (misclassification) across all the decision trees in the forest. Points were allocated to the CT category, asbestos exposure, age, and gender to create a tool for practical clinical use.

## 3. RESULTS

### 3.1. Model performance: Internal testing

**Figure 1.**
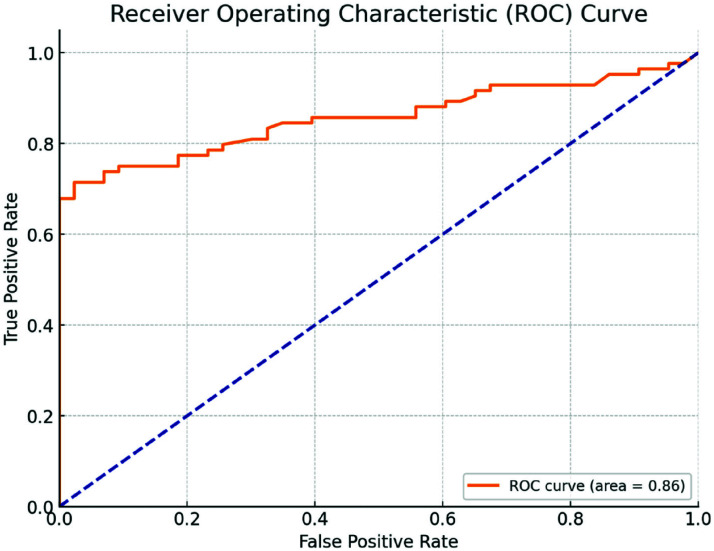
ROC_AUC curve.

The model demonstrated an accuracy of 78%, a precision of 87%, a recall of 79%, and an F1-score of 82%. The ROC-AUC was 0.86, indicating strong discriminative performance (Figure 1).

### 3.2. Model performance: External validation

On the external dataset, the model achieved an AUC of 0.83, with similar precision and recall values, supporting its generalizability. Calibration analysis demonstrated good agreement between predicted probabilities and observed outcomes (Brier score = 0.13).

### 3.3. Feature importance

The features with the highest influence on the predictions were malignant CT findings, asbestos exposure, and age over 60 years. These factors formed the basis of the clinical scoring system (Table 1).

**3.3.1. Score interpretation**

Score ≥ 4: High probability of malignancy. Proceeding directly to tissue diagnosis is recommended.

Score 2–3: Intermediate risk. Standard of care (pleural aspiration) is recommended to rule out infection and attempt cytological diagnosis before invasive biopsy. Tissue diagnosis may still be considered based on clinical judgment.

Score ≤ 1: Low probability. Alternative diagnostic strategies should be considered.

It is crucial to note that pleural aspiration remains the standard for patients with a simplified score of 2–3 (intermediate risk), or for those clinical features suggest infection, such fever or elevated inflammatory markers, which require urgent fluid pH analysis. The bypass pathway is specifically intended for stable patients with a high pre-test probability of malignancy (score ≥ 4).

## 4. DISCUSSION

This study presents the development and external validation of a novel predictive model designed to identify patients with suspected pleural malignancy who may bypass pleural aspiration in favor of direct tissue sampling via thoracoscopy, CT-guided biopsy, or VATS.

It reflects the current literature, which frequently highlights the need for precise tumor subtyping and molecular profiling to guide treatment, as well as the often-reported low yields of pleural aspirations.^11,12^ There is also a group of people for whom, even if cytology is positive, the necessary predictive markers for that tumor cannot be assessed. This “incomplete cytology” therefore necessitates further tissue diagnosis to establish an appropriate treatment plan.^13,14^

Other research has sought to phenotype the clinical predictors of “negative” pleural effusion cytology or “incomplete” cytology—that is, patients with positive cytology but insufficient material to assess predictive markers.^15^ This study highlighted that male gender (p < 0.0001), asbestos exposure (p < 0.0001), and malignant CT findings (p < 0.008) are risk factors for negative pleural cytology. These data mirror our interpretation of predictor importance, providing valuable clinical insight. CT findings, particularly those highly suggestive of malignancy, were the most influential predictors, consistent with the central role of imaging in guiding pleural investigation. Asbestos exposure and age also contributed meaningfully, aligning with known epidemiological risk factors. The inclusion of gender reflects the higher pre-test probability of malignancy in males observed in our cohort and wider literature, likely driven by historical occupational exposure patterns that may not always be explicitly recalled by the patient.

The model demonstrated robust performance, with an ROC-AUC of 0.86 in the internal validation, and preserved a high level of discriminative ability (ROC-AUC: 0.83) when applied to an independent external cohort, thus reinforcing the robustness and generalizability of the approach. A primary strength of this investigation is its external validation. This step, which is frequently absent in AI and clinical prediction model research, provides compelling evidence of real-world applicability and underscores the model’s potential utility across diverse patient populations and healthcare settings.^16^ By applying the model to an external dataset, it demonstrated the model’s resilience to geographic and operational variability, a crucial requirement for future clinical implementation and wider adoption.

### 4.1. Clinical recommendation

The model’s clinical relevance is underscored by its focus on circumventing an often low-yield diagnostic step—pleural aspiration. The British Thoracic Society guideline^5^ recommends aspiration for all new unilateral effusions, but this recommendation predates recent literature that questions its utility in the context of highly suspicious radiological and clinical findings.^3,4^ This model facilitates the stratification of patients for whom aspiration is likely to provide minimal diagnostic value, thereby streamlining care and reducing procedural burden.

This mirrors other research, which is beginning to suggest wider adoption of an approach that proceeds directly to tissue diagnosis in order to shorten time to diagnosis and reduce the number of invasive pleural interventions.^15^ Several studies have demonstrated that time to diagnosis is typically longer in patients with negative pleural cytology, which is associated with repeated pleural procedures,^17–19^ delayed treatment initiation, increased patient anxiety, poorer quality of life, higher complication rates, and reduced overall survival.^20–22^

The simplified scoring system derived from the model offers a pragmatic tool for clinical application to inform earlier decisions to pursue tissue diagnosis. If the required imaging and clinical history are obtained at the time of referral—which could reasonably be expected of a referring clinical—using such a tool could allow earlier planning for diagnostic procedures, potentially even before initial clinic visits. The cut-off of ≥4 for direct biopsy was selected to prioritize high specificity. In this context, a “false positive” (benign pathology undergoing biopsy) carries procedural risks, whereas a “false negative” (malignancy misclassified as low risk) simply defaults the patient to standard care (pleural aspiration), ensuring safety.

This approach aligns with the broader shift in respiratory medicine toward risk stratification and personalized diagnostic pathways. By tailoring the diagnostic pathway to patient-specific risk, clinicians can improve efficiency, reduce the rate of non-diagnostic invasive procedures, and potentially expedite oncological management.

### 4.2. Limitations

Some limitations of the present study warrant acknowledgement. Despite careful curation, the retrospective nature of both the derivation and validation datasets may introduce selection bias. We note the external validation cohort was limited in size; however, the preserved performance metrics support the model’s potential.

Second, while Random Forest models are powerful, they are often considered “black-box” algorithms, which may limit their clinical interpretability.^23^ The lack of transparency in such models can be a barrier to clinical adoption, as physicians may be hesitant to trust a recommendation without understanding the underlying reasoning. To ensure clinical trustworthiness, we considered the interpretability framework proposed by Mediouni et al.^24^ which emphasizes three pillars: feature assessment, traceability, and interaction analysis. By converting our Random Forest output into a transparent scoring system (Table 1), we satisfy the need for traceability, allowing clinicians to see exactly how the risk score is derived. Furthermore, the Gini importance analysis provides robust feature assessment, justifying the heavy weighting of CT findings and asbestos exposure within the model. Future work could further address interpretability by incorporating techniques like SHAP (SHapley Additive exPlanations) values or LIME (Local Interpretable Model-agnostic Explanations) to provide insights into how each variable contributes to the final prediction.^25^

Additionally, our model relies on objective variables available in coded data (age, gender, asbestos exposure, and CT report). We were unable to incorporate subjective variables, such as symptom burden (dyspnoea scores) or precise effusion volume, which may add predictive value but were not consistently recorded in the retrospective dataset.

The study period spans nine years, during which imaging technology and reporting standards may have evolved, potentially introducing temporal heterogeneity into the dataset. Furthermore, the “CT category” variable relies on the semantic interpretation of radiology reports. Inter-observer variability between radiologists was not assessed, which may impact reproducibility across different centres. However, the integration of radiomics—the extraction of quantitative features from medical images—could offer incremental predictive value.^26^ The use of deep learning models, for instance, could analyse these complex image features to predict malignancy with even greater accuracy.^27^

Lastly, although the scoring system simplifies use, it remains a heuristic derived from a complex model, and clinical judgment must continue to guide its application, especially in borderline cases.

### 4.3. Future work

To translate these findings into routine clinical practice, future research must move beyond retrospective validation toward prospective, multi-centre evaluation. A primary focus will be on addressing the sample size limitations of our external cohort by establishing large-scale, prospective observational studies across diverse healthcare settings. This approach will allow testing of the model’s resilience against variations in scanner quality, imaging protocols, and temporal changes in reporting standards over time, ensuring the scoring system remains robust under real-world conditions. Additionally, given the reliance on semantic CT reporting, future studies should assess inter-observer variability between radiologists to ensure that the “CT category” variable is reproducible across different centres.

Furthermore, while this study established diagnostic accuracy, it did not measure downstream clinical impact. Future work must assess whether implementing this “bypass” pathway tangibly improves patient outcomes. Specifically, we aim to design a randomized controlled trial or a pragmatic implementation study comparing the model-guided pathway against standard care. Key primary endpoints will include time-to-diagnosis, time-to-treatment initiation, and the rate of procedural complications, thereby validating the hypothesis that avoiding aspiration reduces morbidity and accelerates care.

Finally, to enhance clinical trust and adoption, future iterations of the model will prioritize explainability. We intend to integrate the model into a decision-support interface within the electronic health record (EHR) for real-time calculation. This development will adhere to interpretability frameworks, such as that proposed by Mediouni et al.^24^ ensuring that clinicians can trace how specific features—such as asbestos exposure or specific CT characteristics—contribute to the individual risk score.

## 5. CONCLUSION

This study presents the development and external validation of a model designed to guide the bypassing of pleural aspiration in favor of direct tissue sampling in patients with suspected pleural malignancy. Demonstrating strong performance across both datasets and practical applicability at the bedside, the model supports a more streamlined, risk-based diagnostic pathway. Crucially, the incorporation of external validation addresses a common shortfall in AI research and enhances the model’s potential for clinical translation.

## COMPETING INTERESTS

The authors have no conflicts of interest to declare.

## AUTHORS’ CONTRIBUTION

AO: Conceptualization, Methodology, Software, Writing—Reviewing and Editing. AG: Data curation, Investigation, Supervision, Resources. AI: Data curation, Writing—Reviewing and Editing, Supervision.

## Figures and Tables

**Table 1.** Predictive clinical scoring system for direct tissue biopsy.

Predictor	Score
CT malignant	+3
CT benign	-2
CT uncertain	+1
Asbestos exposure	+2
Age >60 years	+1
Male gender	+1
